# Kinetic‐Directed Thermodynamic Repair Enables the Synthesis of High‐Strain 2D Sub‐Stoichiometric COFs

**DOI:** 10.1002/advs.76360

**Published:** 2026-06-28

**Authors:** Xiang Pei, Kaifu Yu, Pan He, Hongqing Wu, Bo Jiang, Kewen Shu, Yang Li, Ya Tang, Lijian Ma

**Affiliations:** ^1^ Institute for Disaster Management and Reconstruction Sichuan University Chengdu Sichuan P. R. China; ^2^ College of Chemistry Sichuan University Chengdu Sichuan P. R. China

## Abstract

Two‐dimensional covalent organic frameworks (2D COFs) are promising photocatalysts due to their tunable electronic structures and ordered π‐stacking. Sub‐stoichiometric COFs, featuring periodically unreacted functional groups, offer enhanced property tunability while presenting significant synthetic challenges, especially for strained frameworks like [4+3]‐type COFs. Although previous studies have shown that kinetic conditions strongly influence COF structure, strategies to systematically tune these pathways for precise optimization of crystallinity are still lacking. This work establishes a general kinetic‐controlled strategy wherein modulation of reaction duration or solvent polarity governs the degree of polymerization of kinetic intermediates, directing their subsequent thermodynamic repair into highly crystalline COFs. Using COF‐221 and COF‐222 as models, we demonstrate that varying polymerization time yields amorphous kinetic intermediates with different degrees of polymerization. These intermediates exhibit markedly different crystallization behaviors during thermal repair; only intermediates with an optimal degree of polymerization convert into highly crystalline COFs. Alternatively, employing a high‐polarity solvent (e.g., nitrobenzene) improves monomer solubility and directly produces intermediates with a suitable polymerization degree, effectively merging nucleation and self‐repair into a single step and simplifying what would otherwise require separate kinetic polymerization and thermodynamic repair stages. The resulting COF‐222, achieves efficient charge separation and a 98.6% removal rate in visible‐light‐driven uranium reduction.

## Graphical abstract

2D covalent organic frameworks are promising photocatalysts. Yet, precise synthesis of sub‐stoichiometric COFs remains challenging due to poor kinetic control. We propose a general strategy: tuning polymerization degree of kinetic intermediates via time or solvent polarity directs their thermodynamic repair into highly crystallization. Using [4+3]‐type COF‐222 as a model, this method enhances crystallinity, achieving 98.6% removal rate in visible‐light‐driven uranium reduction.

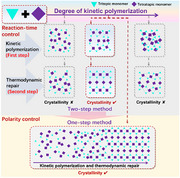

## 1 Introduction

Two‐dimensional covalent organic frameworks (2D COFs) offer significant advantages for photocatalysis owing to their unique extended π‐conjugated and well‐ordered interlayer *π–π* stacked structures [1, 2, 3, 4, 5, 6, 7, 8, 9, 10, 11]. The photophysical properties and catalytic activities of 2D COFs can be finely regulated by precisely controlling the spatial arrangements and electronic structures of their building units at the molecular level, making them ideal design platforms for highly efficient photocatalysts [12, 13, 14, 15]. COFs fabricated using sub‐stoichiometric ratios of monomers are gradually attracting increasing levels of research attention. These materials, which feature periodically unreacted functional groups (such as aldehydo or amino groups), have fully exposed pore domains and polar sites that regulate carrier‐transport pathways, enhance redox activity, and consequently improve photocatalytic performance [16, 17, 18, 19, 20]. Sub‐stoichiometric COFs with the [4+3] topology exemplify this approach: tetratopic monomers function as both ditopic and tetratopic linkers in distinct reaction environments to create frameworks with inherent defects and periodic uncondensed functional groups. These structural modifications significantly change the pore architecture and weaken interlayer stacking, which collectively enhance characteristics of relevance to photocatalysis, ion conduction, and optoelectronics [17].

In contrast to stoichiometric COFs with completely converted linkages, synthesizing sub‐stoichiometric COFs requires controlling selectivities at multiple reaction sites, which makes crystallization particularly challenging. Their syntheses involve highly complex dynamic covalent chemistry that is thermodynamically and kinetically less favorable than that used to prepare conventional COFs, especially for frameworks with inherent strain, as exemplified by [4+3] sub‐stoichiometric COFs comprising tetratopic and tritopic monomers in which concurrent 90° and 120° bond angles generate framework strain that inhibits crystallization. COF synthesis involves multiple steps, including polymerization, nucleation, growth, assembly, and aggregation, the complexity of which challenges crystallization [16, 17, 20, 21, 22, 23, 24, 25]. For example, aldehyde and amine monomers rapidly precipitate as amorphous polymers under acetic acid catalysis during imine COF formation. While these polymers can reorganize into crystalline structures through reversible bond repair under solvothermal conditions, the mechanisms governing their polymerization, nucleation, and growth are poorly understood [21, 24, 25, 26].

The kinetic pathway significantly influences COF crystallization, as demonstrated by Ma et al., who showed that tetra(4‐anilyl)methane and terephthalaldehyde yield COF‐300 with fivefold interpenetration via conventional solvothermal chemistry (120°C, 3 d), and introducing an aging step (3 d at room temperature and 3 d at 50°C) produces a sevenfold‐interpenetrated isomer. This lower‐temperature aging process confers kinetic control over the crystallization pathway [27]. Fischbach et al. further demonstrated that a direct solvothermal reaction for 2 d at 90°C using 3 m acetic acid yields a nonporous, collapsed COF‐300 structure, whereas pre‐reacting the components at room temperature for 2 d, which forms a semi‐reacted intermediate, followed by identical solvothermal treatment, produces porous, crystalline COF‐300^28^. Zhang Jie et al. developed a “freeze–thawing” pretreatment with solvothermal method that optimizes the kinetic pathway toward COF‐366; this approach suppresses excessive monomer polymerization and increases the yield of repairable kinetic intermediates, which ultimately produces well‐defined K‐COF‐366 nanosheets instead of the irregular aggregates (T‐COF‐366) formed conventionally [24].

**SCHEME 1:**
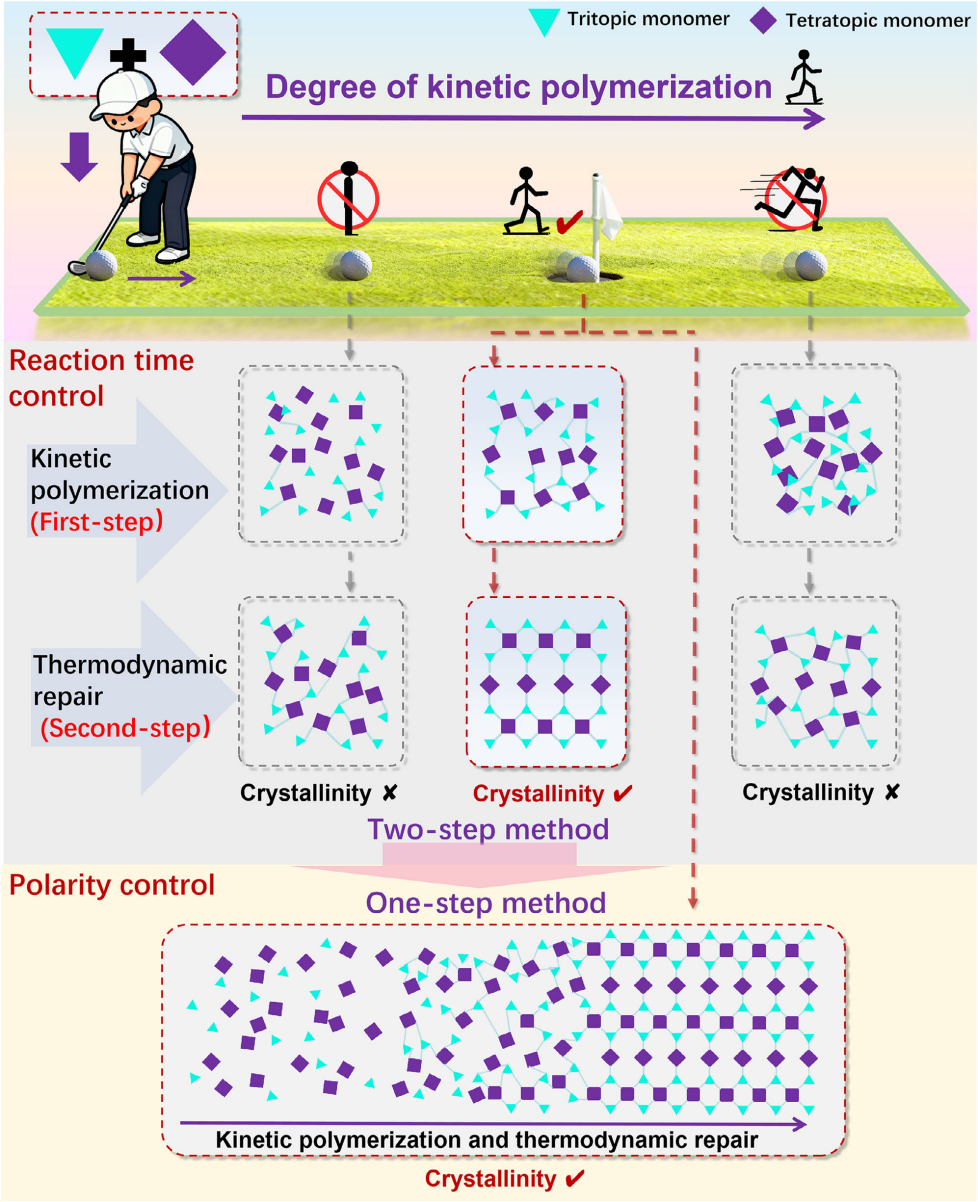
Schematic diagram of controlling kinetic intermediates and their degree of polymerization to promote COF crystallization. Adjusting the reaction time and solvent polarity afford kinetic intermediates that facilitate the formation of highly crystalline COF materials in the subsequent thermodynamic repair stage. Regulating the degrees of polymerization of the kinetic intermediates is akin to a golf championship: only when the right amount of force (degree of polymerization) is applied can the golf ball reach the golf hole of the house (appropriate degree of polymerization) to score and win (crystallization).

The aforementioned examples show that the kinetic pathway critically influences COF crystallization; however, systematic control over crystallization through kinetic regulation remains unexplored. COF growth typically involves fast kinetic polymerization followed by slow thermodynamic repair. Herein, we introduce a strategy that optimizes the polymerization degrees of kinetic intermediates by regulating reaction time and solvent polarity, which facilitates highly crystalline COF formation during subsequent thermodynamic repair. This approach enables control over the kinetic polymerization degree through timed reactions or judicious choice of solvent polarity to promote the development of high crystallinity. We first investigated the effect of reaction time using the two‐step synthesis of COF‐221 from 5,10,15,20‐tetrakis(4‐aminophenyl)‐21H,23H‐porphyrin (TAPP) and 1,3,5‐tris(4‐formylphenyl)benzene (TFPB) as an example. The TFPB aldehyde was first reacted with the TAPP amine under acetic acid catalysis for varying durations to form kinetic amorphous precipitates with various degrees of kinetic polymerization. These precipitates subsequently underwent reversible covalent‐bond repair (thermodynamic repair) under solvothermal conditions, thereby transitioning from a kinetically controlled amorphous state to the thermodynamically stable crystalline COF‐221 structure. The mechanism responsible for the growth of the imine COF was revealed by tracking the structural evolution of the monomer to the crystalline COF by analyzing the intermediates formed, and PXRD (Powder X‐Ray Diffractometer) confirmed the critical roles played by the intermediates in directing crystallization. We further regulated the intermediate polymerization degree through polarity control. Notably, highly polar solvents (e.g., nitrobenzene, which exhibits favorable solubility for TAPP) led to kinetic intermediates with optimal polymerization degrees, thereby enhancing subsequent thermodynamic repair [28]. This method facilitates kinetic polymerization and thermodynamic repair in a unified process (Scheme 1); it produces materials with extended π‐conjugation, mixed quadrilateral/hexagonal pores, and high surface areas when applied to the Benzaldehyde,4,4',4″‐(1,3,5‐triazine‐2,4,6‐triyl)tris‐ (TFPT)–TAPP system (COF‐222) [16, 17, 20, 29]. The triazine units enhance donor–acceptor interactions; furthermore, spectroscopy and computational analysis showed that these units improve charge separation and reduce bandgaps. COF‐222 delivered a 98.6% uranium‐removal efficiency during visible‐light reduction, thereby significantly outperforming COF‐221.

## 2 Results and Discussion

### 2.1 Preparation and Characterization

**FIGURE 1:**
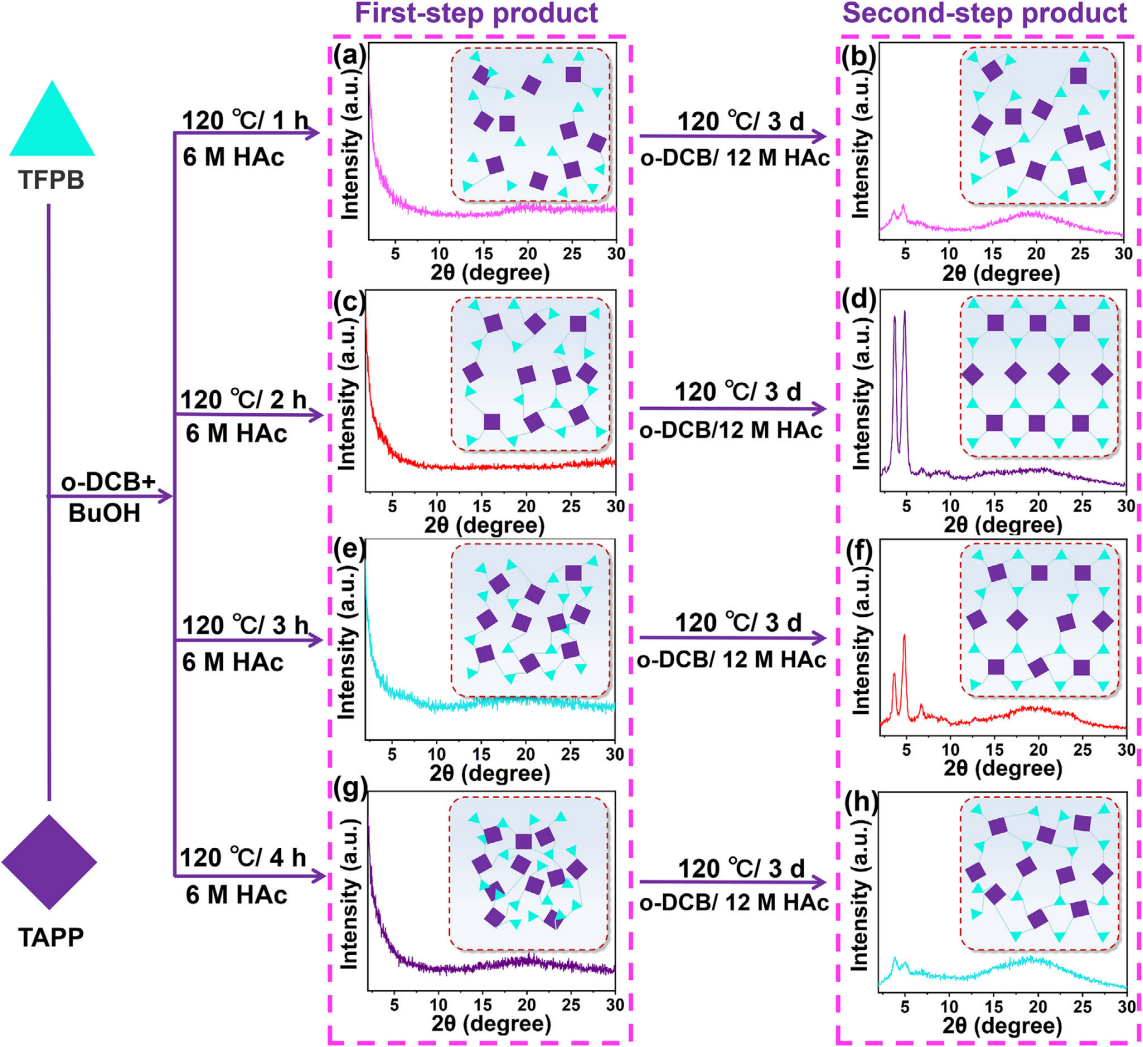
Schematic depicting two‐step methods for the synthesis of COF‐221. Initial products and corresponding PXRD patterns obtained after reacting TAPP with TFPB for (a) 1, (b) 2, (c) 3, and (d) 4 h. Subsequently reactions at 120°C for 3 d afforded second‐step products with the PXRD patterns shown in panels (b), (d), (f), and (h).

A two‐step method was used to investigate how the kinetic pathway impacts COF crystallization. Accordingly, we reacted TFPB and TAPP under acetic acid catalysis at 120°C for varying durations (1, 2, 3, and 4 h), which yielded amorphous irregular precipitates as primary first‐step products (Figure 1a,c,e,g). Subsequent solvothermal treatment at 120°C for 3 d produced distinct second‐step products whose PXRD patterns are shown in Figure 1b,d,f,h. While all first‐step reactions resulted in amorphous intermediates, the second‐step reactions yielded COFs with varying degrees of crystallinity. The kinetic product from a 1‐h first‐step reaction yielded poorly crystalline COF‐221 after the second step (Figure 1b). In contrast, the sample prepared using a 2‐h first‐step reaction produced COF‐221 with the highest degree of crystallinity (Figure 1d). First‐step reaction times beyond 2 h led to clearly less‐crystalline products (Figure 1f,h), with a completely amorphous final product obtained for times exceeding 1 d (Figure S1). These results reveal that the degree of polymerization in the kinetic step significantly influences the efficiency of the subsequent thermodynamic‐repair step and determines the crystallinity of the final COF as a consequence [21, 27, 30]. Therefore, achieving an appropriate degree of polymerization in the kinetic step is essential for producing a highly crystalline COF.

**FIGURE 2:**
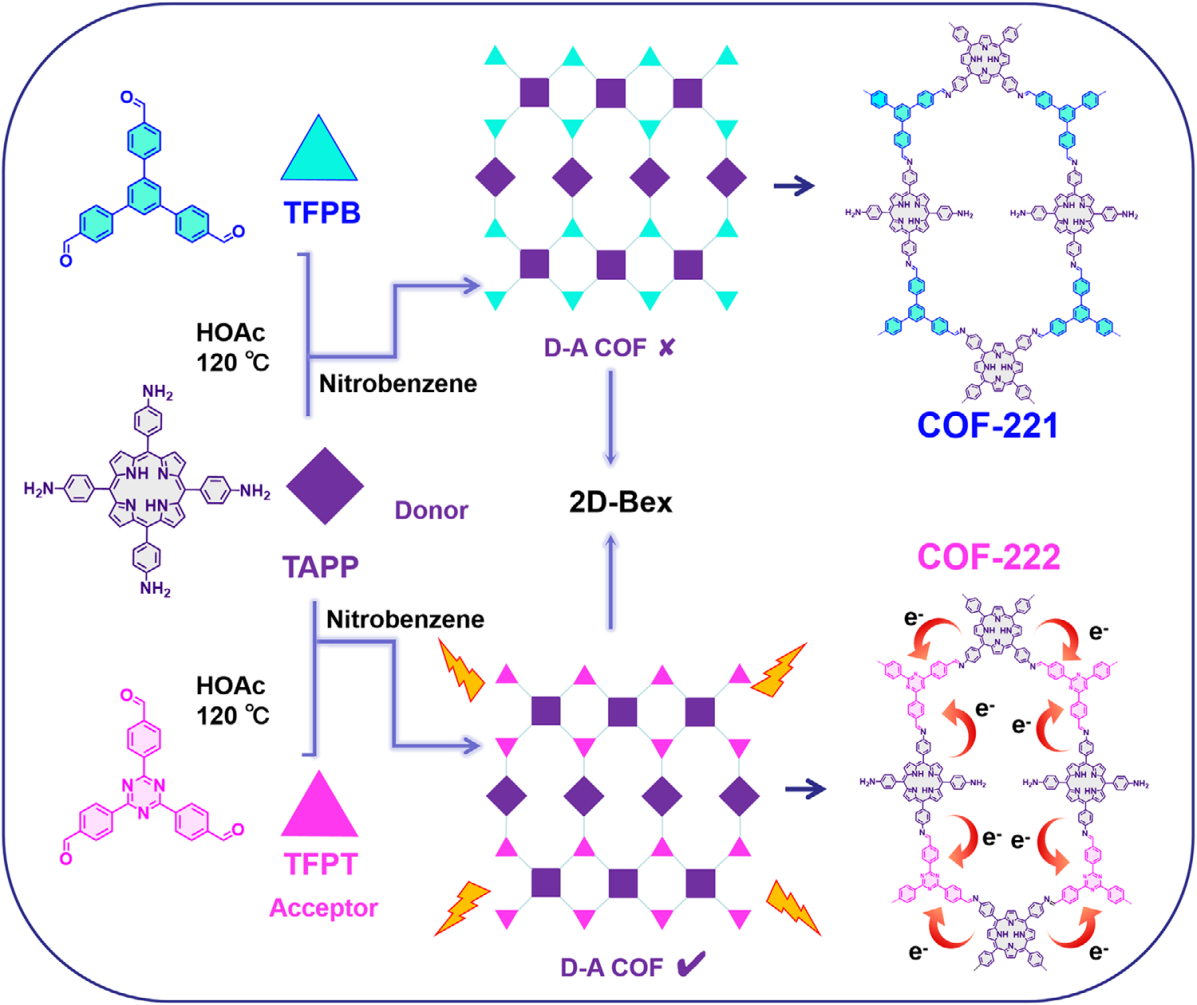
Schematic depicting the syntheses of COF‐221 and COF‐222 with nitrobenzene as the solvent.

The degree of kinetic polymerization is not only influenced by the reaction time but also by the solvent environment. The kinetic polymerization degree is effectively controlled by adjusting the solvent system in a manner that modulates the actual monomer concentration. Based on this principle, we screened solvents to optimize both the kinetic‐polymerization and thermodynamic‐repair steps, examining nitrobenzene (NB), *ortho*‐dichlorobenzene (o‐DCB), *n*‐butanol (BuOH), an o‐DCB/BuOH mixture, dioxane (DOX), mesitylene (Mes), a DOX/Mes mixture, and acetonitrile (ACN). TAPP and TFPB were reacted at 120°C for 3 d in each solvent system, and NB was found to produce the most‐crystalline COF‐221 sample. We systematically measured TAPP solubility in all candidate solvents using UV–vis spectroscopy and established calibration curves to understand how the solvent affects the reaction pathway [26, 31]. TAPP was very insoluble in BuOH, Mes, and ACN (<0.02 mmol/L), while substantially higher solubilities were observed in o‐DCB, DOX, and NB, with NB exhibiting the highest dissolving capacity (Figure S2). We propose that the favorable solubility environment provided by NB favorably regulates the kinetic precipitation process [28] and subsequently facilitates the thermodynamic repair step. This synergy eventually enabled integration of the originally separate kinetic‐polymerization and thermodynamic‐repair steps into a single‐step process. Furthermore, we successfully extended this solvent system to the synthesis of COF‐222 using TFPT and TAPP, which also produced a well‐crystallized material (Figure 2). In contrast, other solvent systems yielded poorly crystallinity in both reactions, as evidenced by PXRD (Figures S3–S5). Notably, in the n‐butanol and o‐dichlorobenzene systems, which exhibited characteristic peaks in PXRD testing, crystalline diffraction peaks emerged in the PXRD patterns. This is likely attributable to the moderate polarity of these solvent systems and their partial solubility for TAPP, thereby facilitating the formation of crystalline features detectable by PXRD. However, the characteristic peaks observed in these systems differed from those of the crystals synthesized in nitrobenzene in terms of signal intensity and full width at half maximum (FWHM). These differences are consistent with the results obtained from our solubility tests for TAPP across the various solvent systems.

**FIGURE 3:**
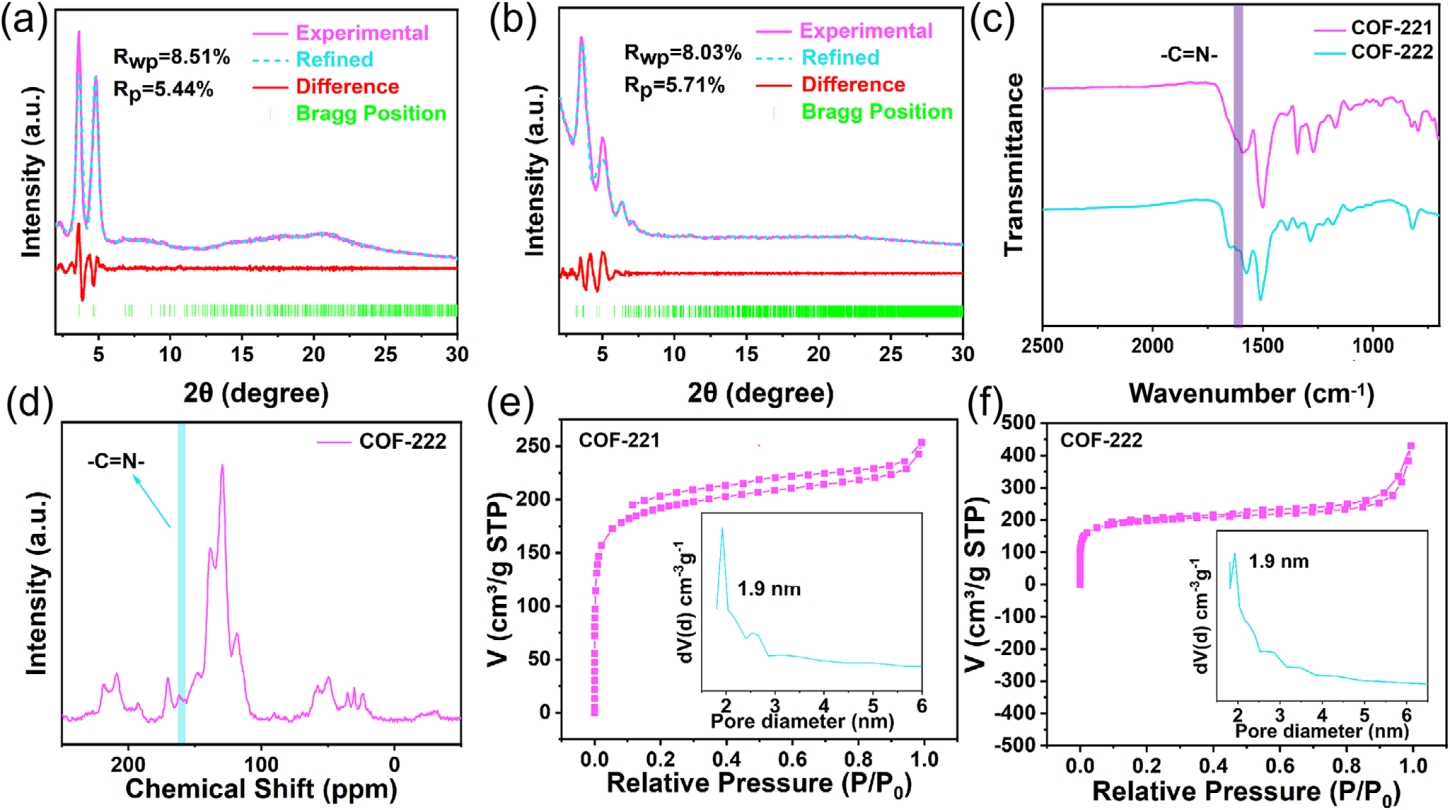
PXRD patterns of (a) COF‐221 and (b) COF‐222. (c) FTIR spectra of COF‐221, and COF‐222. (d) Solid state CP/MAS ^13^C NMR spectrum of COF‐222. N_2_‐adsorption–desorption isotherms and pore‐distributions of (e) COF‐221 and (f) COF‐222.

PXRD confirmed that both COF‐221 and COF‐222 are highly crystalline (Figure 3a,b). Structural simulations using the AB stacking model in Materials Studio revealed PXRD patterns that are in excellent agreement with experimental data (Figure 3a,b, Figures S6 and S7). COF‐221 exhibited characteristic peaks at 3.6° and 4.8° that are highly consistent with the Pawley‐refinement results (Rwp = 8.51%, Rp = 5.44%). Similarly, COF‐222 showed strong peaks at 3.6° and 5.0° that closely match the refined pattern displayed in Figure 3b (Rwp = 8.03%, Rp = 5.71%). FTIR (Fourier Transform infrared spectroscopy) and ^13^C CP‐MAS NMR spectroscopy (^1^ ^3^C Cross Polarization Magic Angle Spinning Nuclear magnetic resonance) confirmed that imine bonds had been successfully formed (Figure 3c,d, Figure S8) [32]. N_2_‐adsorption–desorption experiments at 77 K revealed that both COF‐221 and COF‐222 exhibit typical Type‐IV isotherms (Figure 3e,f). Brunauer–Emmett–Teller (BET) analyses revealed high specific surface areas, while nonlocal density functional theory (NLDFT) pore‐size‐distribution analyses showed that both materials have primary pores with sizes centered at 1.9 nm (Figure 3e,f), consistent with the theoretical structural models. These characterization results collectively confirm that both [4+3] sub‐stoichiometric COFs were successfully synthesized [33].

**FIGURE 4:**
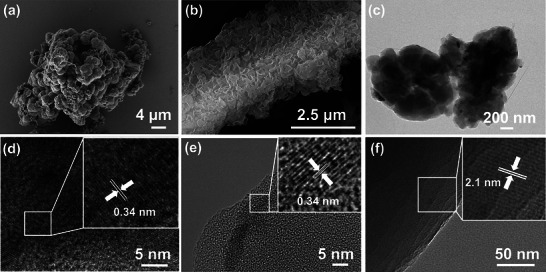
(a) SEM image of COF‐221 and (b) COF‐222. (c) TEM image of COF‐222. TEM images of (d) COF‐221 and (e) COF‐222 showing their layer spacing. (f) TEM image showing the interlayer spacing in COF‐222.

TEM (Transmission Electron Microscope) and SEM (Scanning Electron Microscope) were used to reveal the morphologies and microstructures of COF‐221 and COF‐222. The SEM images in Figure 4a,b show that both materials have distinct, irregular morphologies, while the EDS elemental maps in Figures S9 and S10 confirm that C and N are homogeneously distributed, consistent with their chemical compositions. The TEM images (Figure S11 and Figure 4c) show typical morphological features, with TEM (Figure 4c,d) revealing that both COFs exhibit a lattice spacing of 0.34 nm (Figure 4e). Notably, the lattice fringes observed for COF‐222 by TEM (Figure 4f) further confirm its crystallinity.

**FIGURE 5:**
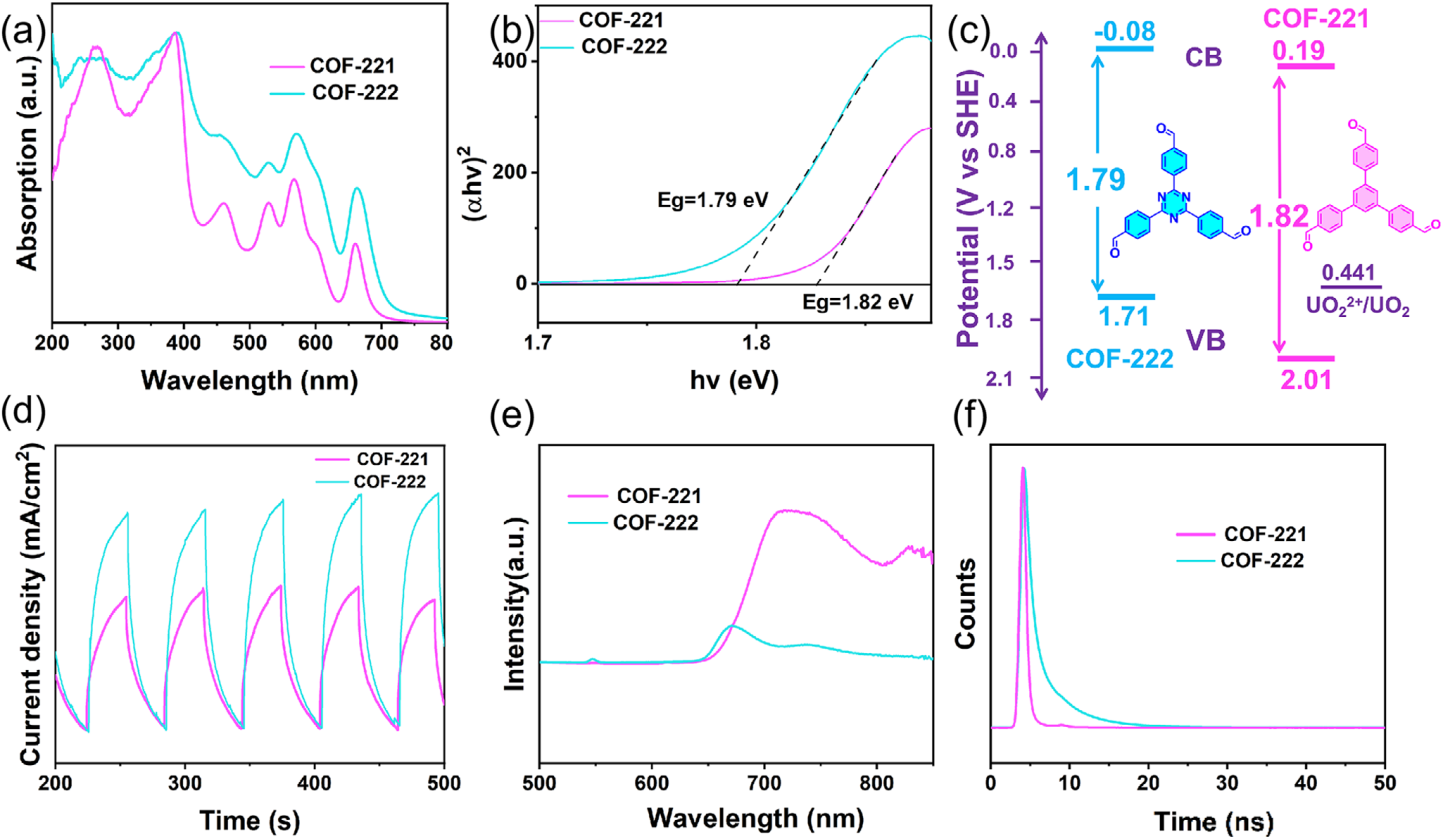
(a) Valence band (VB) XPS spectra of COF‐221 and COF‐222. (b) Bandgaps of COF‐221 and COF‐222 calculated using the Kubelka–Munk transform equation. (c) Energy band diagrams for COF‐221 and COF‐222. (d) Transient photocurrent responses of COF‐221 and COF‐222. (e) Steady‐state PL spectra of COF‐221 and COF‐222 and (f) corresponding temporal decay profiles.

The optical properties and electronic structures of COF‐221 and COF‐222 were investigated by recording their UV–vis diffuse reflectance and steady‐state photoluminescence (PL) spectra. Both COFs absorbed strongly in the UV–vis region, with COF‐222 absorbing significantly more strongly than COF‐221 (Figure 5a). Tauc‐plot analyses yielded bandgaps (E_g_) of 1.82 and 1.79 eV for COF‐221 and COF‐222, respectively (Figure 5b). XPS valence‐band measurements afforded intercepts of 2.25 and 1.95 eV for COF‐221 and COF‐222, respectively (Figure S12), which led to valence band positions of 1.71 and 2.01 eV, respectively, using the equation: E_VB_ (vs. SHE) = WF + E_VB_ (XPS) − 4.44 (using XPS analyzer work function (WF) = 4.20 eV and the standard hydrogen electrode vacuum level of 4.44 eV vs. SHE) [34]. The conduction‐band positions were determined to be −0.08 and 0.19 eV (vs. SHE), respectively, using the relationship: E_CB_ = E_VB_ − E_g_ (Figure 5c). Therefore, the triazine ring linker in COF‐222 delivers a higher conduction‐band position, thereby providing photogenerated electrons of higher energy and enhanced photocatalytic activity.

We next subjected the synthesized COF‐221 and COF‐222 samples to transient photocurrent response experiments (Figure 5d). Reproducible photocurrent responses were observed upon repeated on/off light cycling, which reveals that both COFs effectively capture photons and generate excited‐state carriers, which highlights their potential as photocatalysts. Notably, COF‐222 delivered a higher photocurrent density than COF‐221. Steady‐state PL spectroscopy (Figure 5e) revealed that COF‐222 emits less strongly, which suggests that the triazine ring effectively promotes photogenerated electron–hole‐pair separation. Furthermore, time‐resolved PL spectroscopy revealed that COF‐222 exhibits a longer average emission decay time (τ_n_) than COF‐221 (Figure 5f). Accordingly, the triazine‐containing COF‐222 delivers a higher photocurrent, separates charges more efficiently, and exhibits a longer carrier lifetime than COF‐221, all of which contribute to its remarkable catalytic activity toward the photoreduction of U(VI).

**FIGURE 6:**
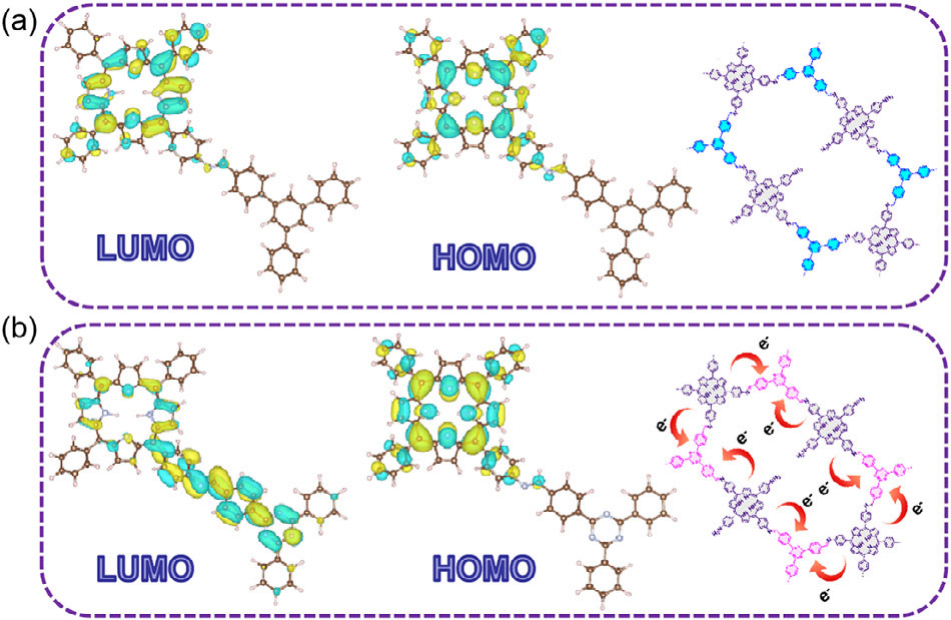
LUMOs and HOMOs of (a) COF‐221 and (b) COF‐222 based on their optimized ground‐state geometric structures.

Charge‐separation behavior was investigated by calculating the electron and hole distributions in COF‐221 and COF‐222 (Figure 6a,b), which revealed that local polarization effects within the COF structures induce significant charge separation [35, 36, 37]. Specifically, TAPP acts as a strong electron‐donating unit and serves as an electron reservoir, while TFPT is electron‐deficient; hence, COF‐222 shows an uneven charge distribution. This observation suggests that TAPP functions as an electron donor, while TFPT is an electron acceptor. Molecular orbital analysis of the COF fragments showed that the HOMO is primarily localized on the donor unit, while the LUMO is distributed across the entire framework fragment. This charge‐density difference confirms the superior charge‐separation capability of COF‐222. In contrast, both the HOMO and LUMO in COF‐221 are localized on the TAPP unit. Consequently, COF‐222, with its strong donor–acceptor (D‐A) structure, separates charge more efficiently than the simple [4+3] sub‐stoichiometric configuration of COF‐221.

**FIGURE 7:**
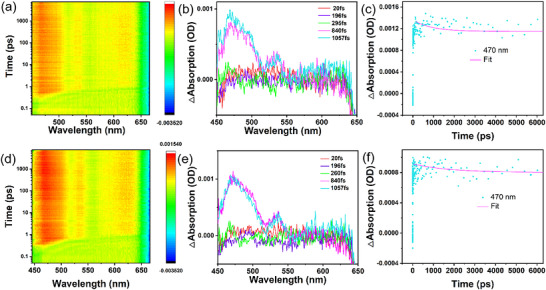
Absorption color maps of (a) COF‐221 and (d) COF‐222, in which the red and blue regions correspond to light‐induced absorption and stimulated emission. (b, e) TA spectra of (b) COF‐221 and (e) COF‐222 acquired at various probe delays. (c, f) Ultrafast kinetics at 470 nm for (c) COF‐221 and (f) COF‐222.

Given the critical role played by photoexcited electron behavior in determining photocatalytic activity, we used femtosecond transient absorption spectroscopy (TAS) with 400‐nm laser excitation to track the photophysical process in each of the synthesized COFs in ethanol (Figure 7a–f). The differential absorption spectra (Figure 7a,d) show red and blue regions that correspond to excited‐state absorption (ESA) and stimulated emission (SE), respectively. COF‐222 absorbs significantly more strongly, indicating that more photoexcited electrons form excited and charge‐separated states in its structure [38]. COF‐222 (Figure 7e) exhibits an initial positive ESA signal at 260 fs (455–520 nm) that corresponds to the excited state, followed by a broad positive absorption at 840 fs (565–630 nm) indicative of a charge‐separated state. In contrast, COF‐221 shows similar states at 296 and 1057 fs, both of which are slightly slower than those observed for COF‐222 (Figure 7c,f) [38, 39]. Moreover, COF‐222 exhibits slower carrier recombination kinetics, the average lifetime of the excited states at 470 nm for COF‐222 was 1750 ps which was much longer than for COF‐221 (1445 ps) [3, 39, 40]. Accordingly, charge‐transfer efficiency follows the order: COF‐222> COF‐221.

**FIGURE 8:**
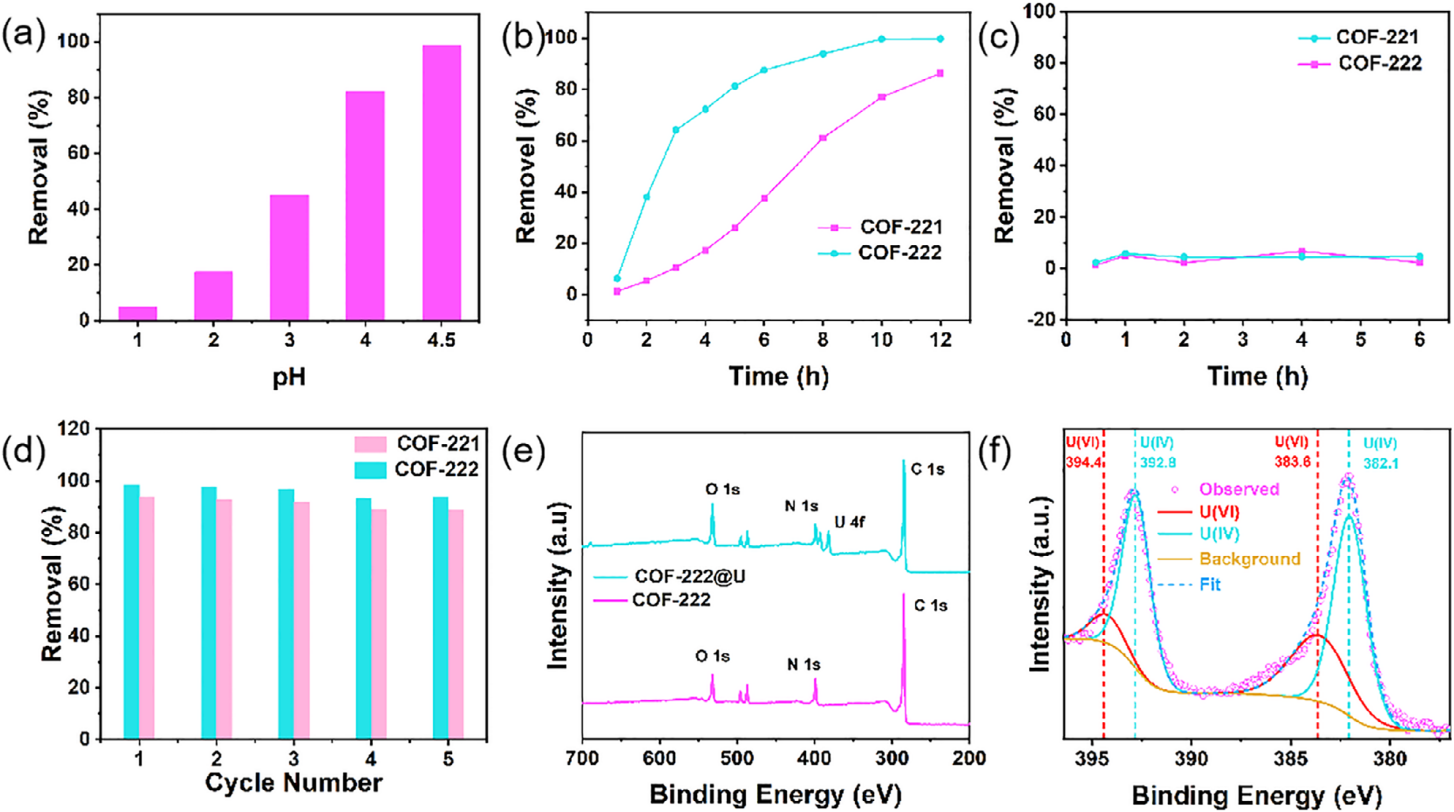
(a) Effect of solution pH on the removal of uranium by COF‐222 (C_0_ ≈ 50 mg/L, t = 12 h, m = 10 mg, v = 20 mL). (b) Uranium‐removal rates of COF‐221 and COF‐222 at pH 4.5. (c) Uranium‐removal ratios of COF‐221 and COF‐222 under dark condition. (d) Uranium‐removal ratios of COF‐221 and COF‐222 during five successive rounds of use. (e) XPS spectra of COF‐222 and COF‐222@U. (f) Deconvoluted U 4*f* XPS spectrum of COF‐222@U.

Given the unique photoelectronic properties of COF‐222, we next investigated its performance in photocatalytic uranium reduction. This study commenced by investigating the effect of solution pH. Figure 8a shows that COF‐222 removes uranium in a pH‐dependent manner, with removal increasing with rising pH to its optimal value at pH 4.5. This trend is attributable to protonation under highly acidic conditions, which imparts a positive surface charge that electrostatically repels uranyl cations (UO_2_^2^^+^), thereby lowering the removal efficiency. Supporting this hypothesis, zeta potential measurements in the 1–4.5 pH range confirm that increasing the pH reduces the positive charge (Figure S13). COF‐222 removed 98.6% of the uranium at pH 4.5, which is significantly higher than that recorded for COF‐221 (Figure 8b); it also exhibited high selectivity toward uranium compared to other metal ions (Figure S14). Both materials exhibited minimal uranium uptake in adsorption experiments conducted in the dark (Figure 8c), which confirms that the removal process is dominated by photocatalytic reduction [29]. Both COFs were highly stable over five 12‐h reaction cycles (Figure 8d) [41]. The uranium‐extraction mechanism was elucidated by analyzing uranium‐loaded COF‐222 (COF‐222@U) following the photoreaction. The XPS survey spectra displayed in Figure 8e reveal the emergence of U 4*f* peaks, indicative of uranium adsorption [42, 43]. The high‐resolution U 4*f* spectra displayed in Figure 8f show pairs of peaks at 394.4/383.6 and 392.8/382.1 eV that correspond to U(VI) and U(IV), respectively, which confirms that U(VI) is reduced to U(IV) during photocatalysis.

## 3 Conclusion

This study investigated precisely controlling COF crystallinity by systematically regulating the kinetic pathway via an innovative synthesis strategy. The novel approach centers on modulating the polymerization degrees of intermediates via reaction time or solvent polarity to promote the formation of a highly crystalline framework during subsequent thermodynamic repair. This concept was validated for the two‐step synthesis of COF‐221, which involves the rapid formation of amorphous intermediates followed by their solvent/thermal conversion into crystalline products via reversible bond reorganization, via PXRD and other techniques, confirming the critical role played by the intermediates. Solvent screening revealed that regulated polarity solvents such as nitrobenzene exhibits favorable solubility, thereby controlling kinetic intermediates with “appropriate polymerization degrees” and facilitating the merger of nucleation and self‐repair into a single step. The strategy was further extended to COF‐222, which retained the characteristic mixed‐pore architecture, high surface area, and extended conjugation of a [4+3] substoichiometric COF while exhibiting enhanced light absorption and more‐pronounced donor–acceptor (D‐A) characteristics. These features contributed to the superior performance of COF‐222 during the photocatalytic reduction of uranium, thereby delivering 98.6% removal rate at an initial uranium concentration of 50 mg L^−^^1^ at pH 4.5, which significantly surpasses the performance of COF‐221. This study not only expands synthetic methodology for the formation of COFs but also demonstrates how designing D‐A structures at the molecular level in substoichiometric frameworks can lead to optimal photoactive materials, thereby offering new pathways for solar‐energy conversion and advancing the practical applications of covalent organic frameworks.

## Author Contributions

**Hongqing Wu**: investigation. **Bo Jiang**: investigation. **Yang Li**: supervision, investigation, conceptualization. **Xiang Pei**: conceptualization, investigation, funding acquisition, writing – original draft, methodology, validation, visualization, writing – review and editing, software, formal analysis, project administration, data curation, resources. **Pan He**: methodology. **Kaifu Yu**: data curation. **Lijian Ma**: supervision. **Kewen Shu**: methodology. **Ya Tang**: supervision.

## Conflicts of Interest

The authors declare no conflicts of interest.

## Supporting information




**Supporting File**: advs76360‐sup‐0001‐SuppMat.docx.

## Data Availability

The data that support the findings of this study are available from the corresponding author upon reasonable request.
