# Supplementary material for: Kinetic‐Directed Thermodynamic Repair Enables the Synthesis of High‐Strain 2D Sub‐Stoichiometric COFs
Source: Adv Sci (Weinh). 2026 Jun 28:e76360. Online ahead of print. doi: 10.1002/advs.76360 (PMC13336355; doi:10.1002/advs.76360)
Supplement: Supplementary file 1 — Supporting File: advs76360‐sup‐0001‐SuppMat.docx. [file ADVS-9999-e76360-s001.docx]

Supporting information

*Xiang Pei^a^, Kaifu Yu^b^, Pan He^b^, Hongqing Wu^b^, Bo Jiang^b^, Kewen Shu^b^, Yang Li^b*^, Ya Tang^a^ and Lijian Ma^b*^.*

*^a^ Institute for Disaster Management and Reconstruction, Sichuan University, Chengdu, Sichuan 610064, PR China*

*^b^ College of Chemistry, Sichuan University, Chengdu, Sichuan 610064, PR China*

**Materials and Methods**

*Chemicals and Materials*: Experimental Reagents: methanol, ethanol, N,N- dimethylformamide, o-dichlorobenzene, and n-butanol were purchased from Chengdu Changlian Chemical Reagent Co., Ltd. 5,10,15,20-tetra(4-aminophenyl)porphyrin (TAPP) was purchased from Energy Chemical Technology (Shanghai) Co., Ltd. 1,3,5-Tris(4-aminophenyl)benzene (TFPB) was purchased from Energy Chemical Technology (Shanghai) Co., Ltd. 4,4',4''-(1,3,5-Triazine-2,4,6-triyl)trianiline (TFPT) was purchased from Energy Chemical Technology (Shanghai) Co., Ltd. All reagents and materials used were of high purity and were purchased directly from commercial suppliers without the need for further purification.

**Characterization and Method**

The powder X-ray diffraction (PXRD) patterns were obtained using a Bruker D2 phaser diffractometer. The morphology of COFs was characterized by scanning electron microscopy (ZEISS Gemini 300). ^13^C solid-state NMR spectra were performed on a Bruker 400M spectrometer. The elemental chemical states on the surface of materials were measured by X-ray photoelectron spectroscopy (Thermo Scientific K-Alpha). Fourier transform infrared spectra (FTIR) were recorded by a PerkinElmer IR-843 spectrometer (USA). Transmission electron microscopy (TEM) images were performed on a JEOL JEM F200 instrument at the accelerating voltage of 200 kV. The fs-TA measurements were performed employing a regenerative amplified Ti: sapphire laser system (coherent; 800nm,85 fs, 7 mJ pulse-1, and 1 kHz repetition rate) as the laser source and a Helios spectrometer (Ultrafast Systems LLC), as described previously.

**Syntheses**

*Preparation of COF-221 by two steps method:* Fist-step*:*COF-221 was synthesized by condensing TAPP (15 mg, 0.025 mmol) and TFPB (8.8 mg, 0.033 mmol) with 6 M acetic acid as a catalyst (0.1 ml) in 0.6 ml 1,2-dichlorobenzene and 0.15ml 1-Butanol at 120 °C over a period of 2hours. Second step: the products of the above reactions were subjected to a 120 °C over a period of 3 days reaction with 12 M acetic acid as a catalyst (0.1 ml) in 1,2-dichlorobenzene(0.2 ml), respectively.

*Preparation of COF-221 and COF-222 with Nitrobenzene*: COF-221 was synthesized by condensing TAPP (15 mg, 0.025 mmol) and TFPB (8.8 mg, 0.033 mmol) with 12 M acetic acid as a catalyst (0.1 ml) in 0.2 ml Nitrobenzene at 150 °C over a period of 3 days. The reaction was performed in a 10 ml Pyrex tube with nitrogen atmosphere. The resulting black precipitate was filtered, washed, and dried, giving a yield of approximately 91%. COF-222 was synthesized by condensing TAPP (15 mg, 0.025 mmol) and TFPT (12 mg, 0.033 mmol) with 12 M acetic acid as a catalyst (0.1 ml) in 0.2 ml Nitrobenzene at 150 °C over a period of 3 days. The reaction was performed in a 10 ml Pyrex tube with nitrogen atmosphere. The resulting black precipitate was filtered, washed, and dried, giving a yield of approximately 83%.

**Uranium Enrichment Tests**

A specific quantity of UO_2_(NO_3_)_2_•6H_2_O was dissolved in acidic water containing 0.1 mol/L HNO_3_ to formulate the U(VI) solutions. The pH level of the U(VI) solution was adjusted to 4.5. A glass reactor was filled with a 50 mg/L solid-to-liquid ratio of a 0.5 mmol/L U(VI) solution under ambient conditions, without the addition of any sacrificial agents. Immediately, an air atmosphere was maintained while illuminating the glass reactor with a Xenon lamp (Perfectlight PLS-SXE300) that delivered an intensity of 200 mW cm^−2^. To test the reusability of COF-221 and COF-222, after the photocatalysis experiment, the photocatalysts were collected and subjected to ultrasound in 50 mL of 0.1 M NaHCO_3_ or 1M HNO_3_ solution for 3 h. Subsequently, the photocatalysts were washed with deionized water for the subsequent cycle. Furthermore, the photocatalytic tests were carried out in 0.2 mM U(VI) solutions with varying pH levels. For the ion interference test, 0.5 mM U(VI) solution and 0.5 mmol/L interfering ions solutions were used. The concentration of U(VI) was analyzed by inductively coupled plasma-atomic emission spectrometry (ICP-OES) after solid-liquid separation. In order to test the reusability, the photocatalysts were collected after the above photocatalysis experiment and stirred in 50 mL of 0.1 M NaHCO3 solution for 2 h. Then, deionized water was further used to wash the photocatalysts for the next cycle. The U(VI) removal ratio (%), U(VI) extraction capacity (mg/g), and extraction rate constant (k) were determined as [(C_0_-C)/C_0_] × 100%, (C_0_-C) × V/m, and -ln (C/C_0_) = kt, respectively. In this calculation, C0 represents the initial concentration of U(VI) (mg/L), C represents the final concentration of U(VI) (mg/L), t represents the reaction time (min), V represents the solution volume (L), and m represents the sample mass (g).

The distribution coefficient can be calculated by the following formulas:

$$K_{d}=\frac{\left( c_{0}-c_{e} \right)\times v}{mc_{e}}$$

where c_0_ and c_e_ are the initial and equilibrium concentrations (mg/L). v (L) represents the volume of used solution, and m (g) is the weight of COF powder.

*Theoretical Calculations*: All the PXRD model was stimulated by Material Studio (MS). The LUMO and HOMO of COF-221 and COF-222 were investigated using the Projector Augmented Wave (PAW) potentials with Perdew-Burke-Ernzerhof (PBE) of exchange correlation interactions via the Vienna Ab-initio Simulation Package (VASP). Using the conjugate gradient algorithm for structural relaxation, the self-consistent energy convergence threshold was set to 1×10^–5^ eV, and the ionic convergence criterion was -0.01 eV/Å. The Monkhorst-Pack scheme was used to set the k grid as 1×7×1 to sample the Brillouin zone. We selected the lowest energy band in the conduction band and the highest energy band in the valence band as the LUMO and HOMO, respectively, and visualized them using vaspkit.


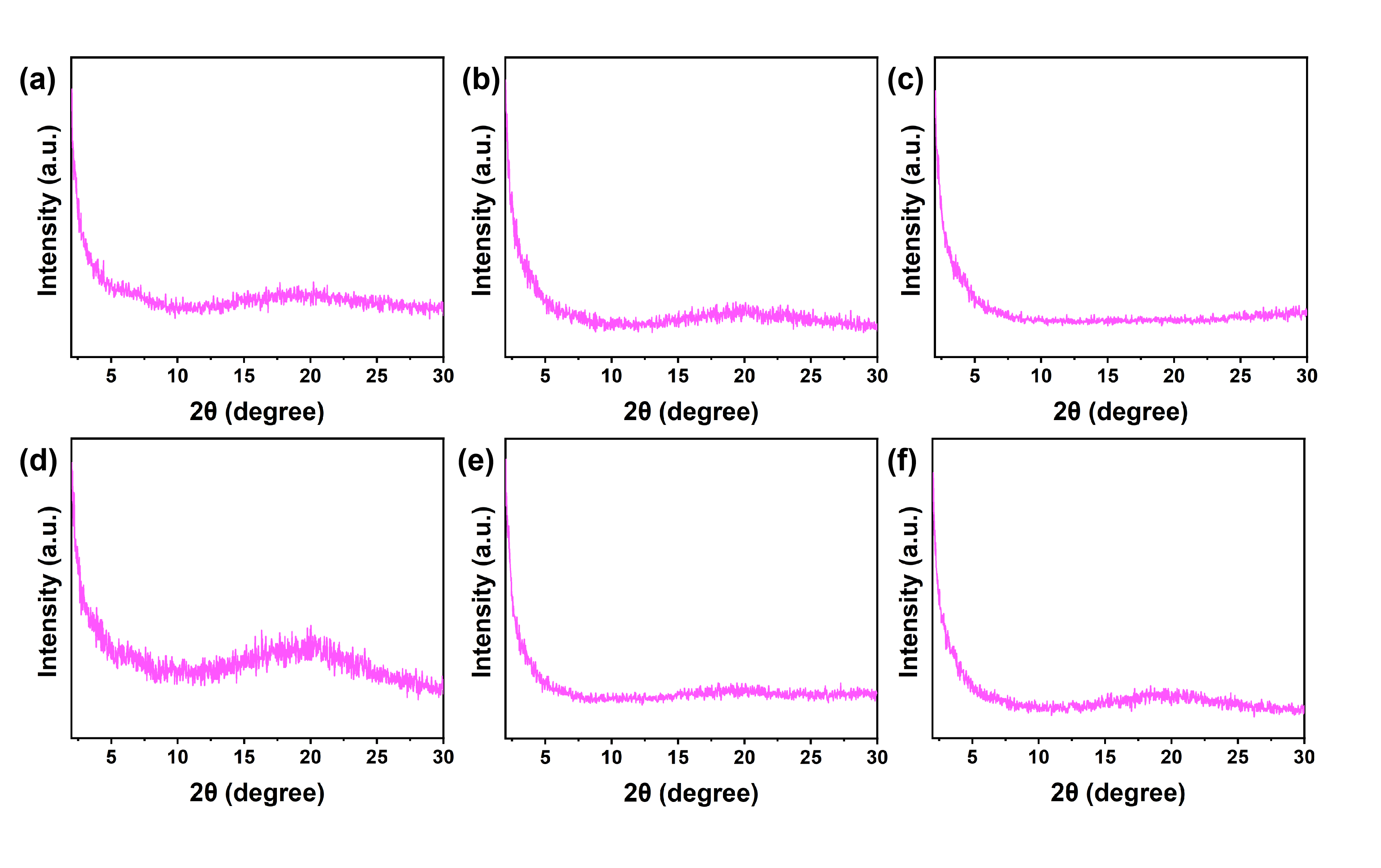


Figure S1. Schematic of the syntheses of COF-221 via two-step method. The first-step product and thier PXRD formed from the reaction after (a)1d, (b)2d and (c)3d undergoes a further reaction at 120°C for three days to yield the second-step product and PXRD (d), (e) and (f).


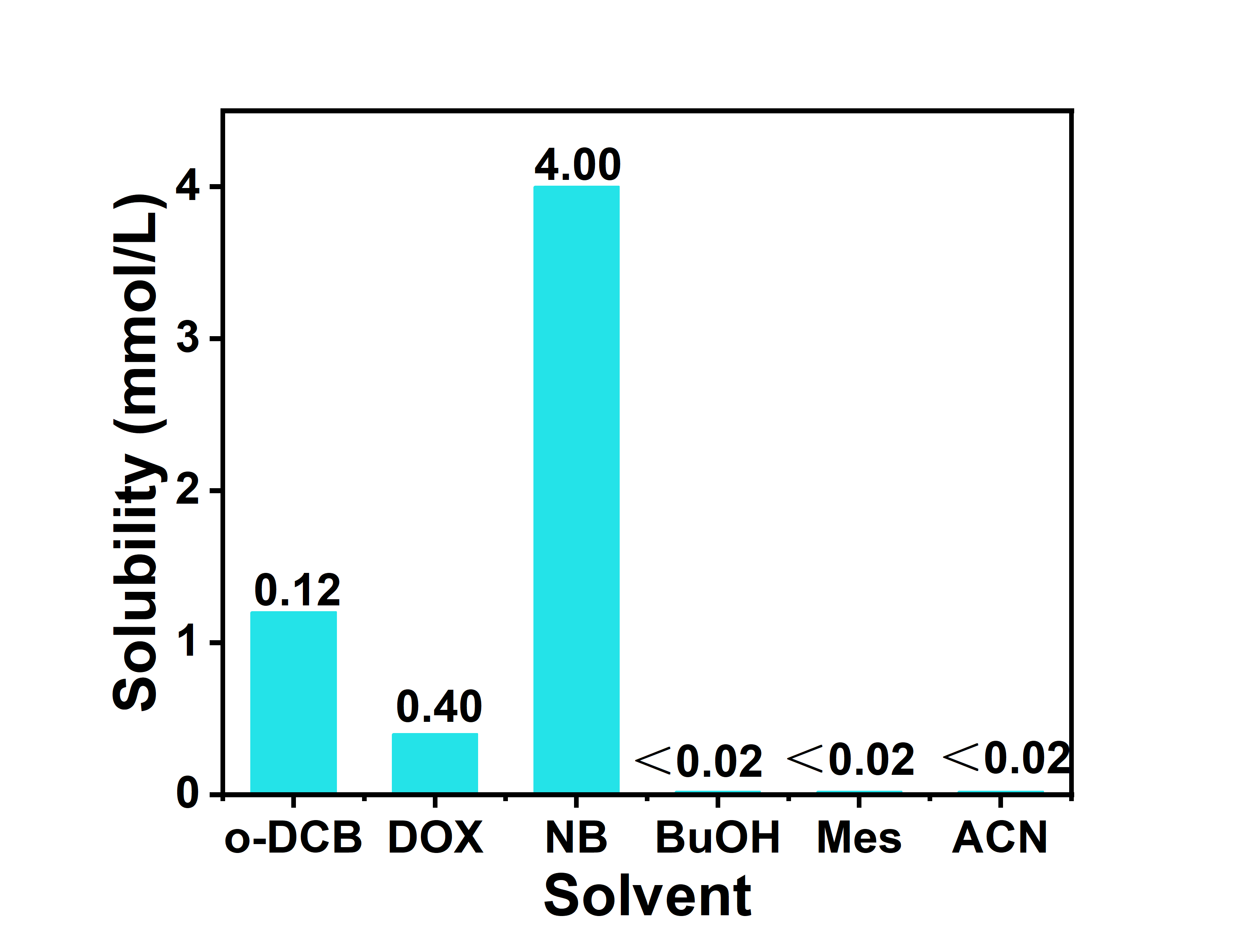


Figure S2. Saturation solubility of TAPP in o-Dichlorobenzene (o-DCB), 1,4-Dioxane (DOX), Nitrobenzene (NB) n-Butanol (BuOH), mesitylene (Mes), and acetonitrile (ACN).


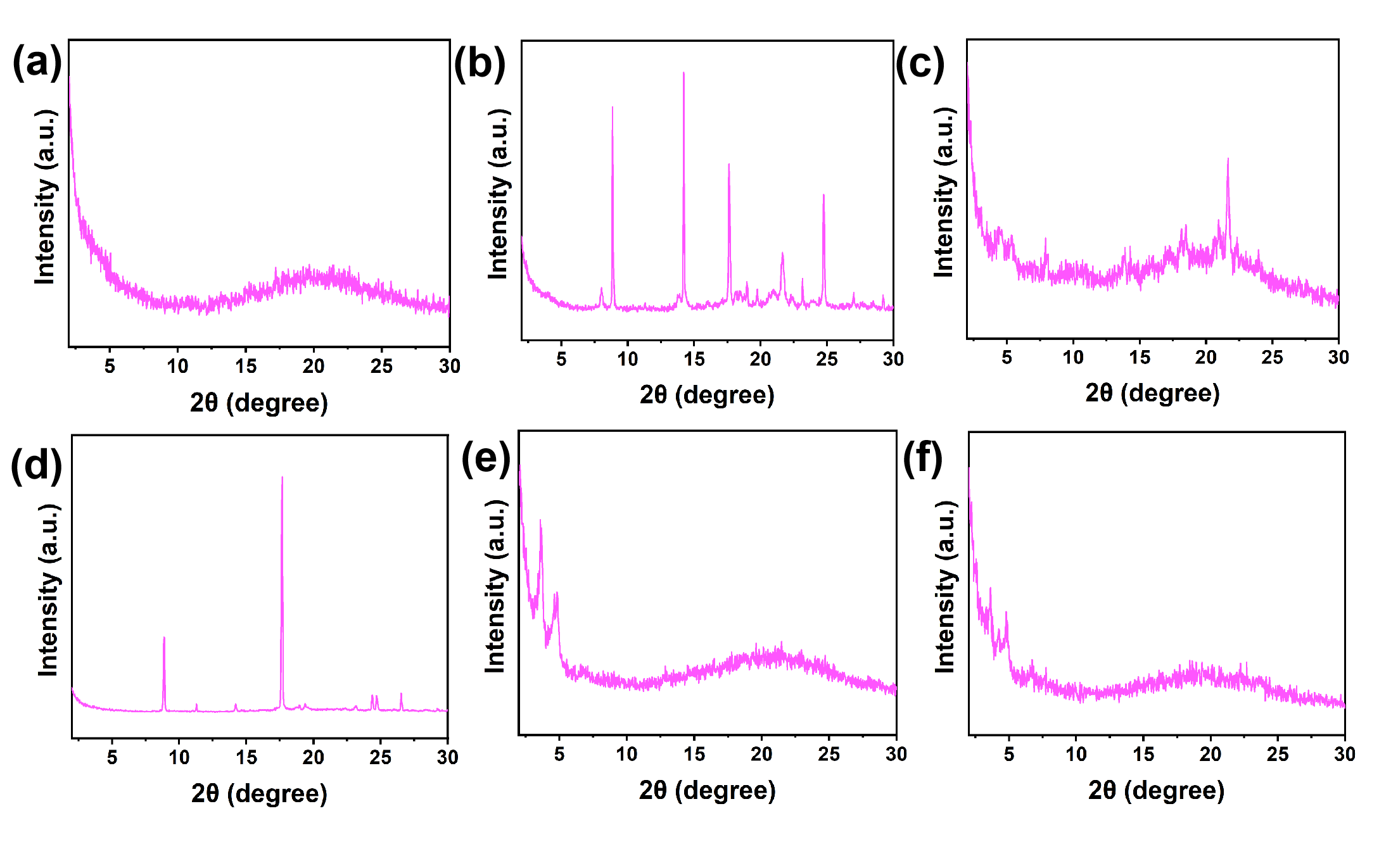


Figure S3. Powder X-ray diﬀraction patterns of COF-221 synthesis in (a) dioxane, (b) 1,3,5-Trimethylbenzene (c) dioxane with 1,3,5-Trimethylbenzene (d) o-Dichlorobenzene, (e) n-butanol and (f) o-Dichlorobenzene with n-butanol.
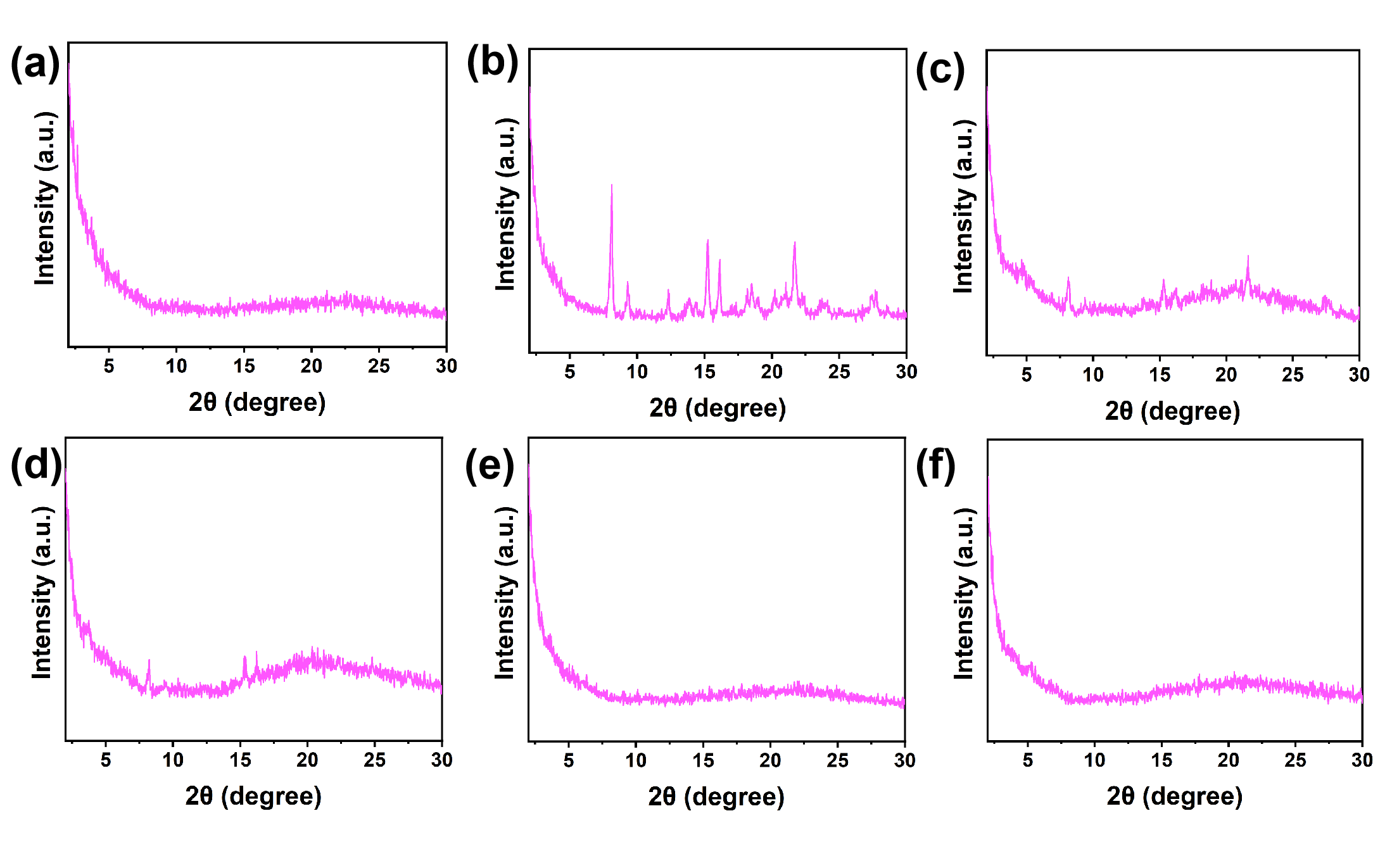


Figure S4. Powder X-ray diﬀraction patterns of COF-222 synthesis in (a) dioxane, (b) 1,3,5-Trimethylbenzene (c) dioxane with 1,3,5-Trimethylbenzene (d) o-Dichlorobenzene, (e) n-butanol and (f) o-Dichlorobenzene with n-butanol.


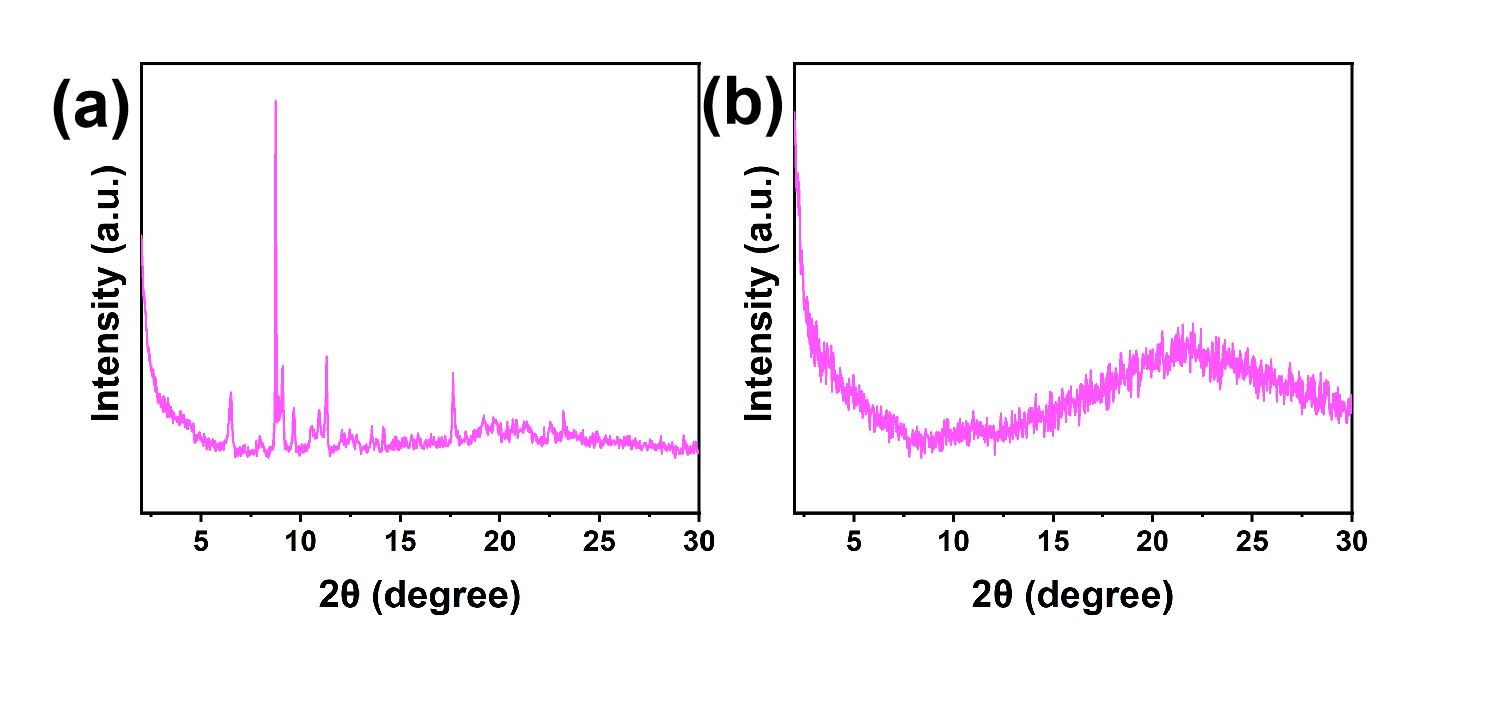


Figure S5. X-ray diﬀraction patterns of (a) COF-221 and (b) COF-222 synthesis in acetonitrile.


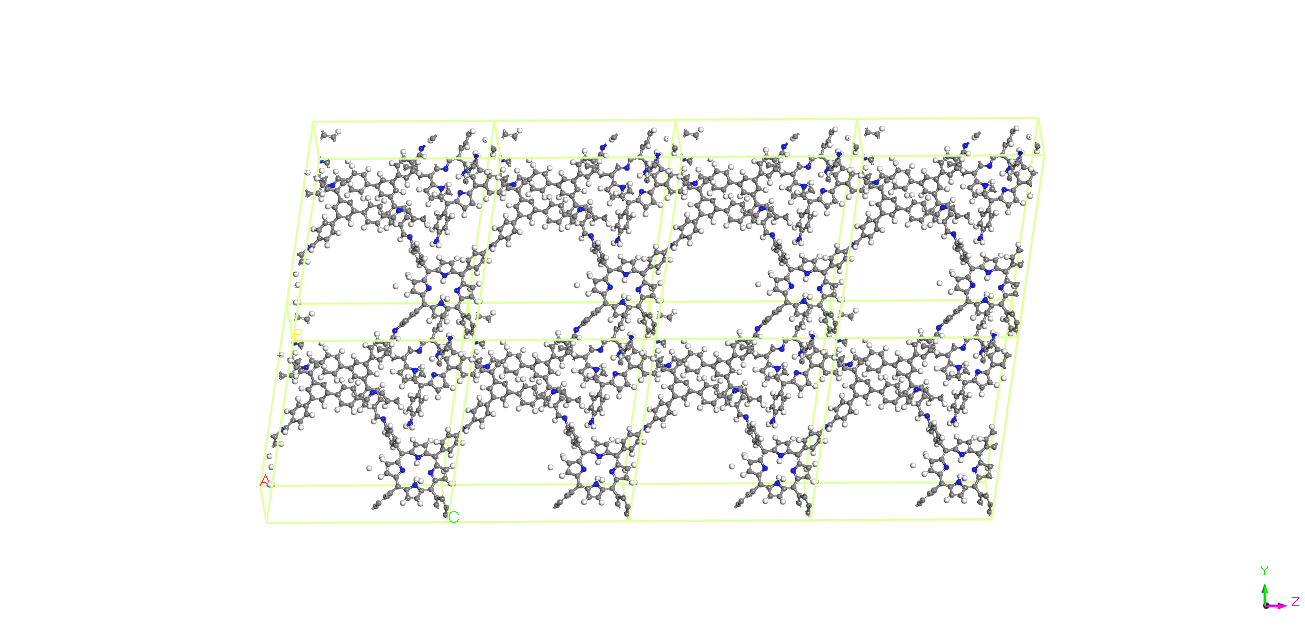


Figure S6. Stimulation models of COF-221 by Materials studio.


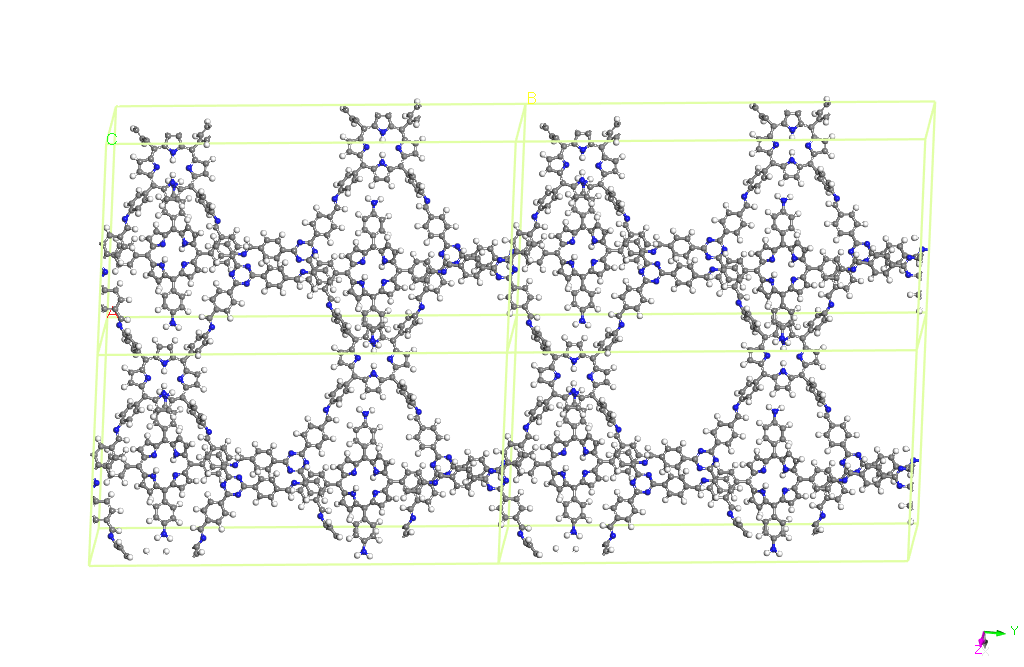


Figure S7. Stimulation models of COF-222 by Materials studio.


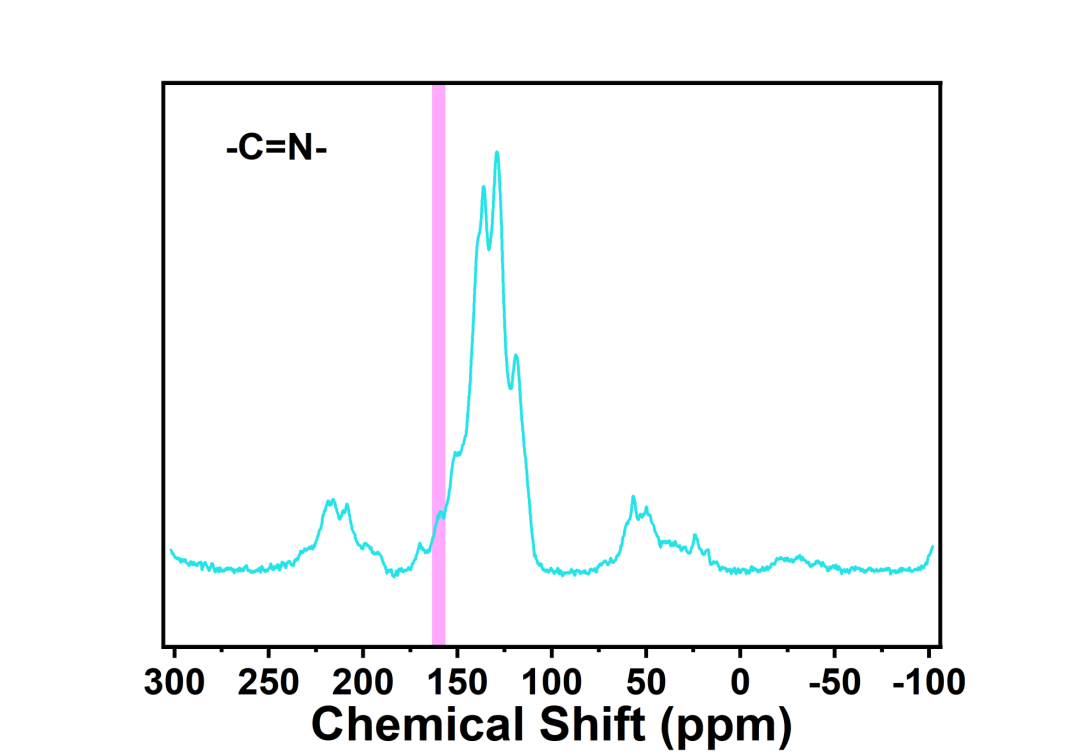


Figure S8. Solid state CP/MAS ^13^C NMR spectrum of COF-221.


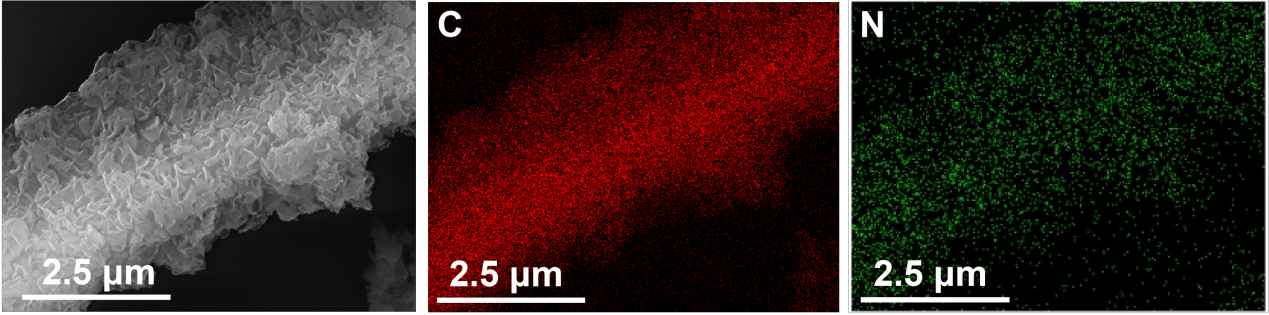


Figure S9. The corresponding elemental mapping images including C and N of COF-222.


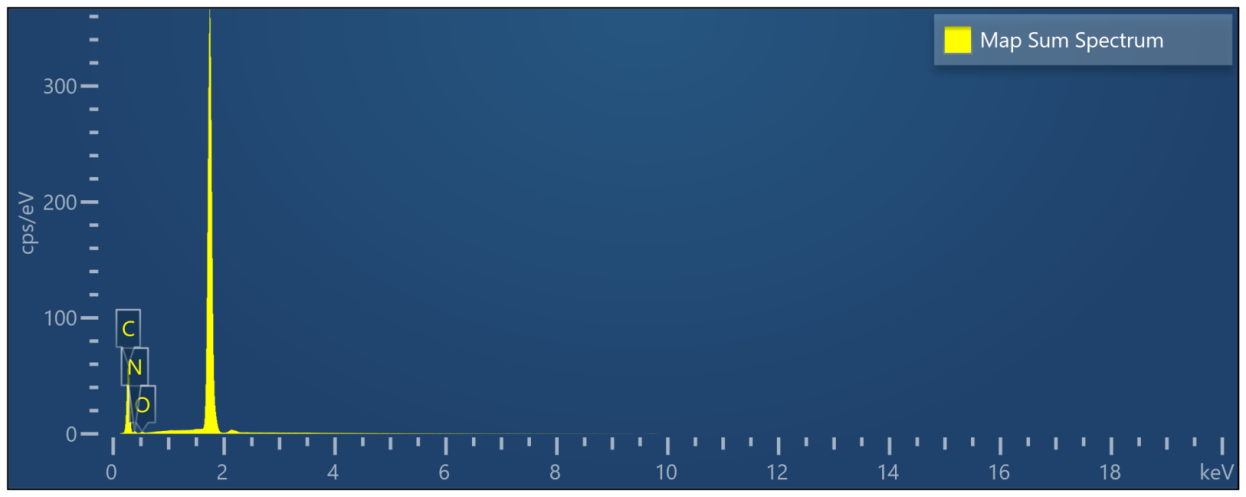


Figure S10. The corresponding elemental content of COF-222.


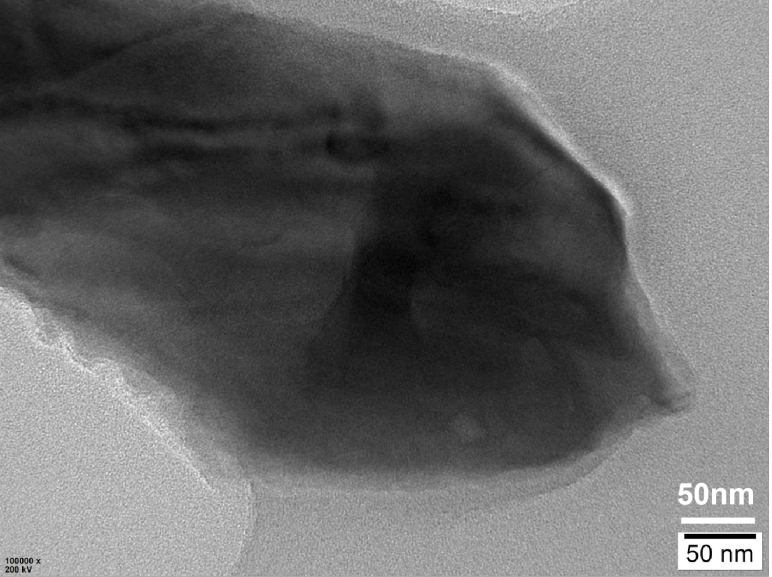


Figure S11. TEM images of COF-221.


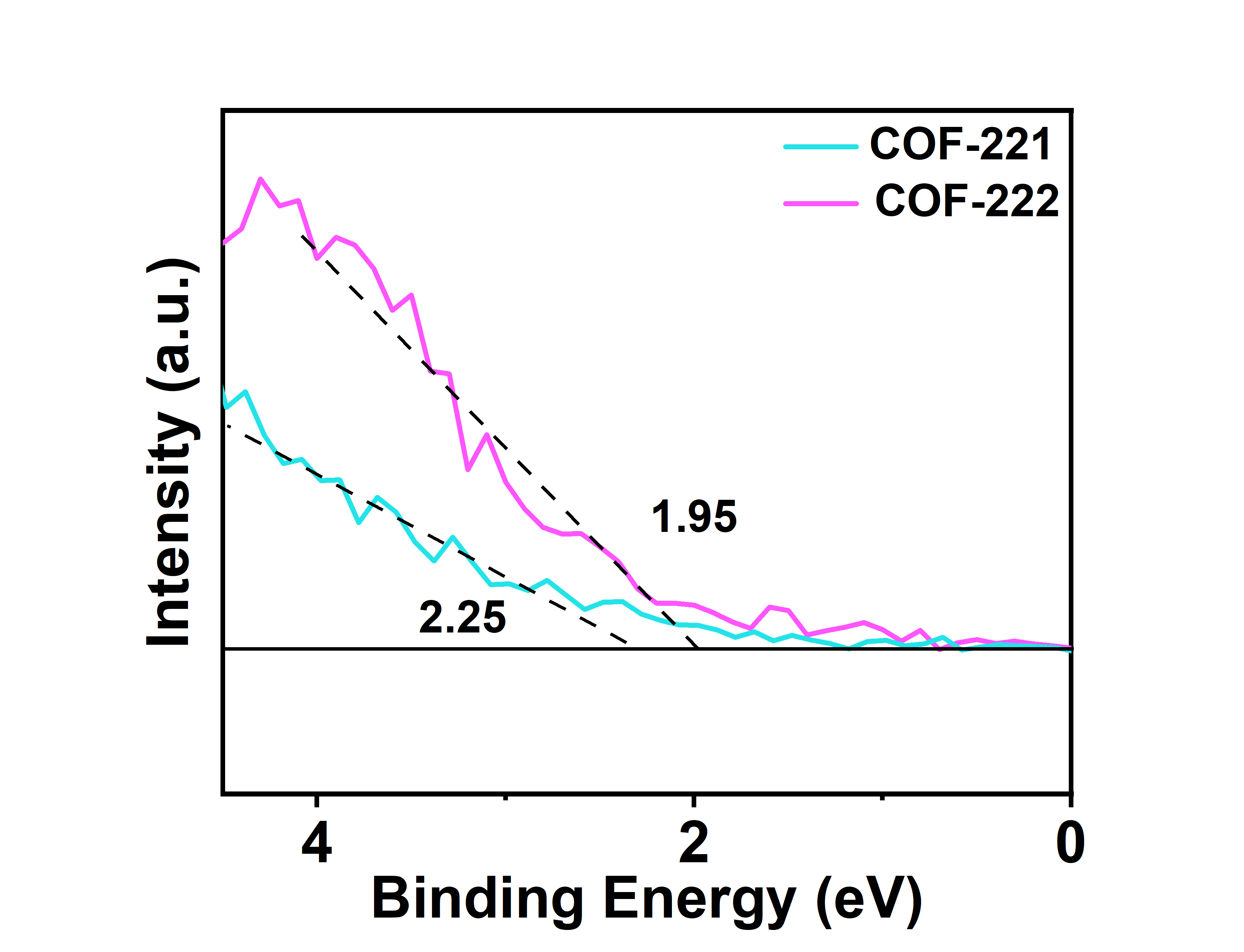


Figure S12. UV-Vis DRS of COF-221 and COF-222.


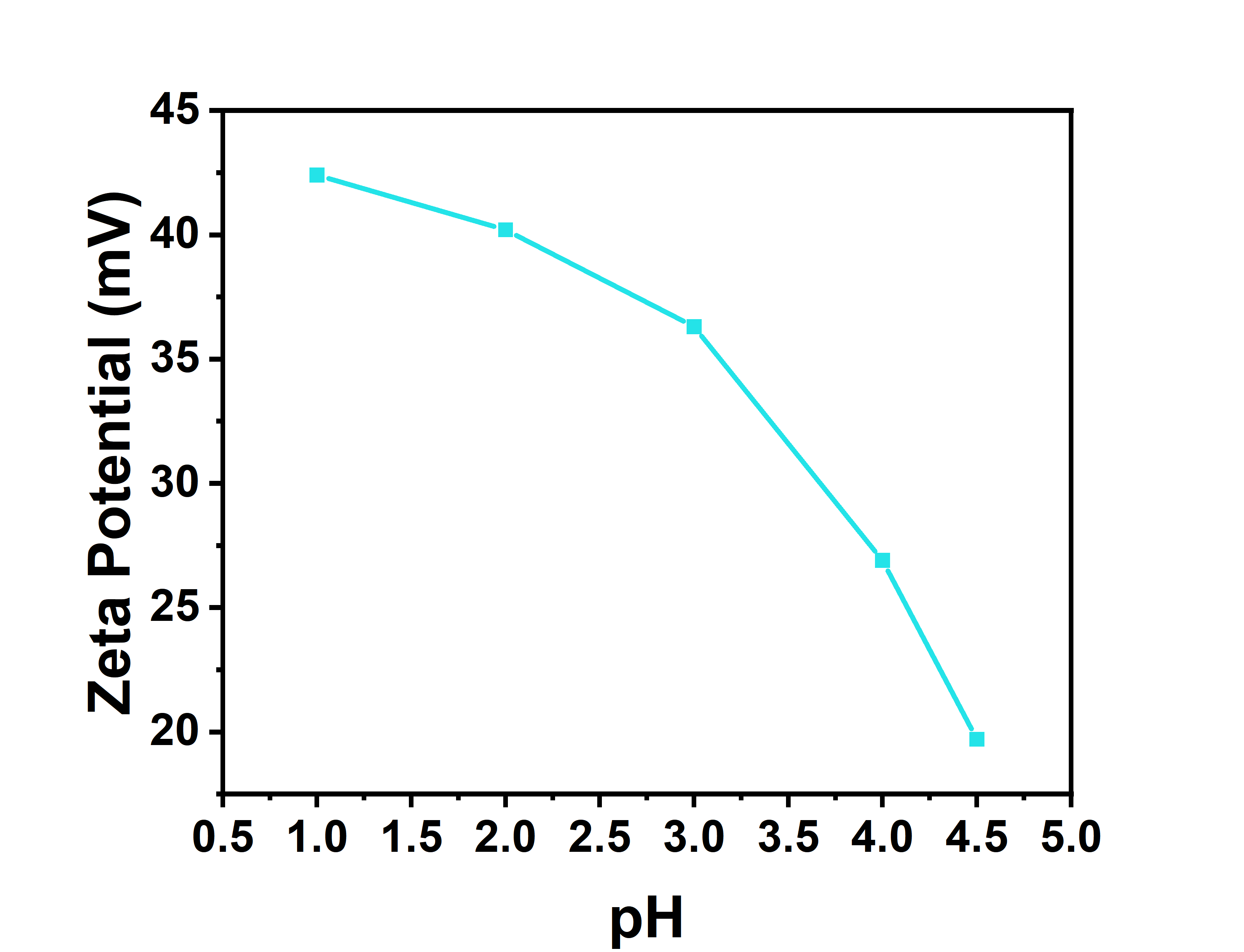


Figure S13. Zeta potential of COF-222 in different pH values.


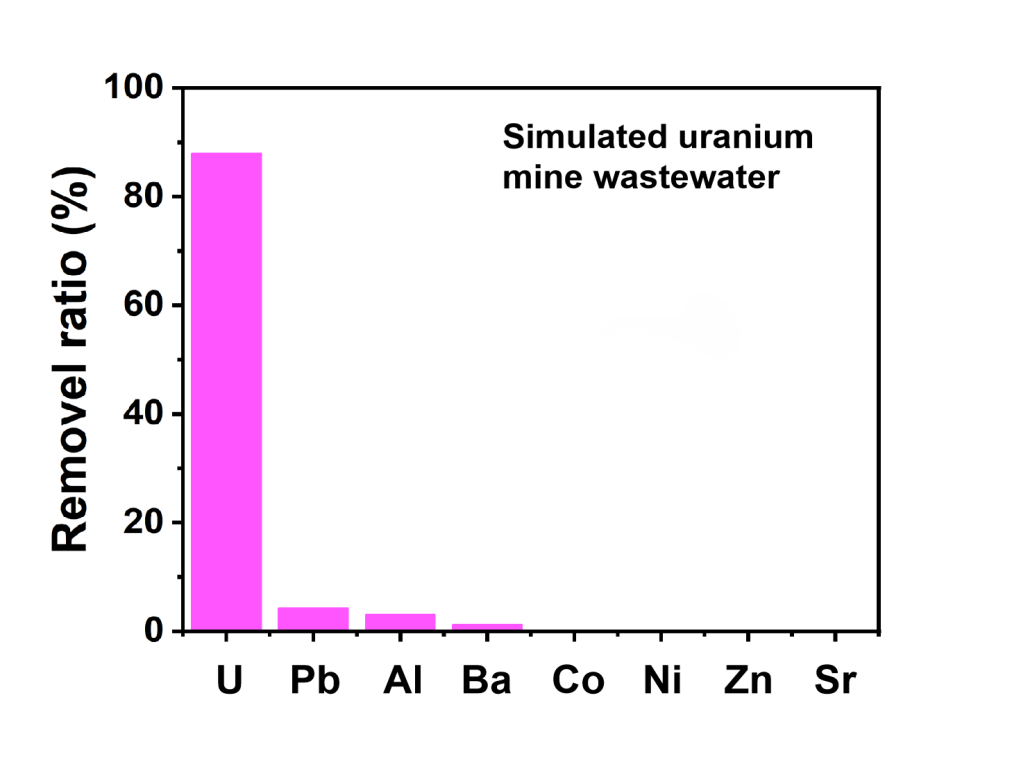


Figure S14. Removal rate via COF-222 in the uranium solution with 11 cations.
